# Novel evaluation method based on critical arch height as instability criterion for sustaining arch locked-segment-type slopes

**DOI:** 10.1038/s41598-024-58737-w

**Published:** 2024-04-05

**Authors:** Lijin Wang, Hang Jia, Tong Jiang, Junran Zhang, Yanchang Jia, Longfei Li, Li Wan

**Affiliations:** 1 College of Geosciences and Engineering, North China University of Water Resources and Electric Power, Zhengzhou, 450046 China; 2 Zhejiang Huadong Geotechnical Investigation & Design Institute Co, Ltd, Hangzhou, 310014 China

**Keywords:** Landslide, Model test, Locked-segment, Sustaining arch, Critical arch height, Instability prediction and early warning, Natural hazards, Engineering

## Abstract

In sustaining arch locked-segment-type slopes, natural soil arches play a key anti-sliding role in the slope's evolution. In this study, a self-developed model test device was used to simulate the whole process of deformation evolution of sustaining arch locked-segment-type slopes, and the formation of natural sustaining arch and its locking control effect on slope stability were studied. The test results show that the continuous formation and progressive destruction of the sustaining arch were observed. The sustaining arch formed in the second time has the best locking effect, and the anti-sliding force reaches its stress peak point. However, the slope is not in a critically unstable state, instead, the stress is continuously adjusted to form a larger range of soil arch to resist the slope thrust. Consequently, the slope destabilizes until the ultimate shear strength of arch foots is exceeded, at which point the critical arch height of the arch is reached. The critical arch height mechanical model for slope stability analysis was developed based on the soil arching effect and limit equilibrium theory. The applicability of the model was demonstrated by the physical test and Xintan slope data, which can provide some guidance for early warning of landslides.

## Introduction

The locked segment refers to the unpenetrated part that plays a key bearing role on the potential sliding surface of the slope and is subject to stress concentration, for example rock bridges, retaining walls, and sustaining arches^1–3^. During the sudden destruction of the locked segment, the accumulated high energy results in a high-speed remote landslide, often resulting in huge casualties and economic losses^4–7^. Therefore, it is extremely urgent to prevent and mitigate the disasters caused by such slope instability. Locked-segment-type slopes are more common in nature, especially in accumulation slopes where sustaining arch structures can be easily formed. In the study of the Xintan slope in the Three Gorges Reservoir area, Wang et al.^8^ proposed that the Jiangjiapo area may form a “sustaining arch” across the slope owing to the basement uplift; this was deduced based on the deformation signs before the landslide. In succession, scholars have confirmed the existence of the natural soil arching effect in the Xintan landslide, the Lizi Township landslide and the Maijianwo landslide by means of planar optical stress measurement analysis and numerical simulation^9–12^. Cheng et al.^13^ confirmed that the stability of such slopes is primarily determined by the slope's geometric conditions, boundary conditions, and mechanical properties. However, they did not analyze the relationship between the progressive damage of the arch and the evolution of slope instability. Fang et al.^14^ proposed a simplified geomechanical model to analyze the deformation characteristics of such slopes. Chen et al.^3^ confirmed that the sustaining arch is the key internal factor controlling the stability of the Xintan slope by mechanical analysis, and explained why the Xintan landslide was not triggered by the maximum rainfall time. Although a lot of research has been accumulated around the geological, mechanical and physical basis of this type of landslide. However, the research on the sustaining arch locked-segment-type slopes is not deep enough, the complete development process and physical–mechanical mechanism of the natural sustaining arch are not clear, and the relationship between the progressive destruction process of the sustaining arch and the evolution of slope instability needs to be further clarified.

For the sustaining arch locked-segment-type slopes, the instability mechanism is more intuitive and clear, and the slope stability is highly predictable. Current landslide stability prediction methods are mainly based on phenomenological, statistical or empirical predictions. However, this statistical prediction only evaluates the association between landslides and related parameters, not the failure mechanism of landslides^15–17^, which has a high level of subjective arbitrariness, resulting in many disasters not being foreseen. Therefore, in order to make significant progress in landslide prediction research, it is necessary to shift from empirical prediction to physical prediction, starting from the perspective of physical and mechanical mechanism and evolution process^18–21^. For sustaining arch locked-segment-type slopes, the natural soil arch structure formed by stress redistribution plays a key anti-sliding role in the overall stability of the slope body^3^. Therefore, more attention should be paid to when the soil arch structure disintegrates, which may enable more accurate predictions to be made.

The natural soil arching effect in geotechnical engineering is defined as a spontaneous phenomenon where the stress transfers from the yielding soil mass to the adjoining unyielding soil^21–24^. At present, considerable research has been conducted on the soil arch produced by embankment pile foundation structures^25,26^ and slope support structures in semi-infinite soil^27–31^. Researchers generally believe that the arching effect is determined by various factors, such as load^25^, pile spacing^28,32^, pile configuration^30^, slope angle^33^, embankment height^25,26^, soil conditions^27,31^, etc. Zhang et al.^31^ reported that the formation and evolution of the soil arching effect of an anchor anti-sliding pile can be roughly divided into three stages; in these stages, the arch height continuously increases with the destruction and re-formation process of the soil arch. For the natural soil arch in geotechnical engineering, when the sliding force increases to a certain value, the soil arch will increase the thickness of the arch ring to resist a greater load^34^. The compression zone behind the pile also increases, but the shear and compressive capacity of the soil limits the deformation of the soil arch^28,35^. When the foot of the slope reaches the ultimate shear strength, the sustaining arch cannot continue to form, and the soil arching effect fails. At this time, the height of the arch reaches the critical arch height. Therefore, for the sustaining arch locked-segment-type slopes, the arch height of the sustaining arch should be an important element for real-time monitoring and early warning.

In this study, a self-developed model test device was used to simulate the whole process of deformation evolution of the sustaining arch locked-segment-type slope. As a result, the continuous formation process of the sustaining arch was observed and its progressive destruction process was revealed. Furthermore, considering the evolution and physical–mechanical mechanism of the slope, the critical arch height prediction model for evaluating the stability of slopes is established by combining the limit equilibrium theory and soil arching effect. And the applicability of the model was verified by the model test results and the Xintan landslide data. The results show that the critical arch height of the sustaining arch locked-segment-type slopes is a function of the geometric parameters of slope, the geometric parameters of the arch support and the geotechnical parameters. The research results are of great significance to improve the stability analysis, instability prediction and early warning of this type of slope.

## Geological structure characteristics of typical sustaining arch locked-segment-type slopes

### The Xintan landslide

**Figure 1:**
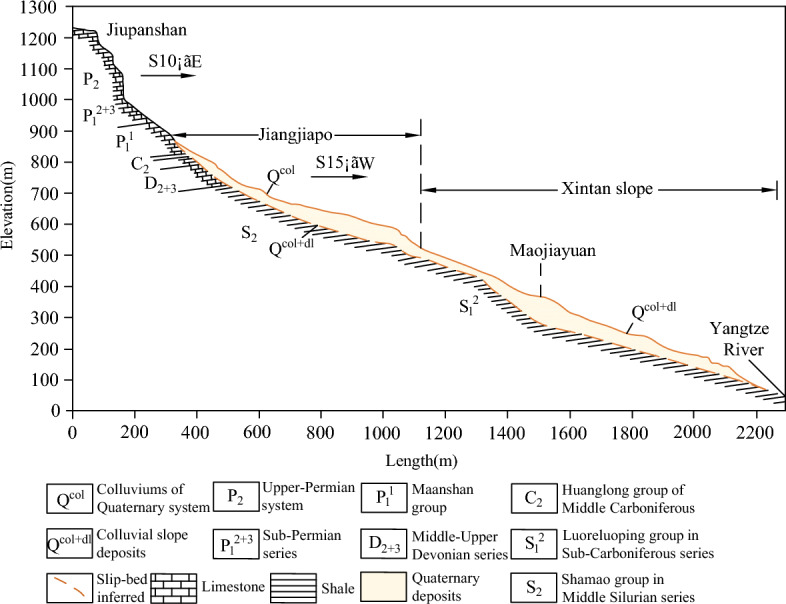
Longitudinal profile of the landslide study area (modified from references^38,39^).

A more typical sustaining arch locked-segment-type landslide is the Xintan landslide that occurred on June 12, 1985, on the left bank of the Yangtze River, 27 km upstream from the Three Gorges Dam. The Xintan landslide was developed based on the old landslide, and the accumulation of back edge loads and slope material was the underlying cause of the sliding of the Jiangjiapo slope^3,36,37^. From the signs of the pre-slip slope deformation, a 'sustaining arch' across the slide may have existed in the Jiangjiapo area due to the raised base, narrowing on the west side and coinciding with the bend on the east side^8^ (Fig. 1). The arch abutments on both sides are the important joint parts of the upper and lower body, and also the part where the soil is squeezed and compacted.

It is worth noting that the surface of the soil on the east and west sides of the Jiangjiapo slope was wet prior to the slide. This may be because of the groundwater seeping out of the ground as a result of the deformation of the soil blocking the outlet points in the bedrock, or it may be due to the pore water being squeezed out by the rapid compression of the soil, causing a local rise in the groundwater table. Both these circumstances can increase the pore water pressure in the soil, thus reducing the shear strength of the soil and the contact surface between the soil and the bedrock^8^. If the soil liquefies owing to a sudden and significant increase in super-pore water pressure, its shear strength is significantly reduced. At this point, the sudden reduction in shear strength of this part of the soil may destabilize and cause damage to the arch support and foot of the soil arch, resulting in sliding of the upper section of the side slope (Jiangjiapo slope). Therefore, the sliding mechanism of this landslide is mainly a sliding of the upper part, supported by the central "arch", and locked in place by the arch support. The western end of the arch slides and breaks down, resulting in an overall destabilization.

### The Maijianwo landslide

**Figure 2:**
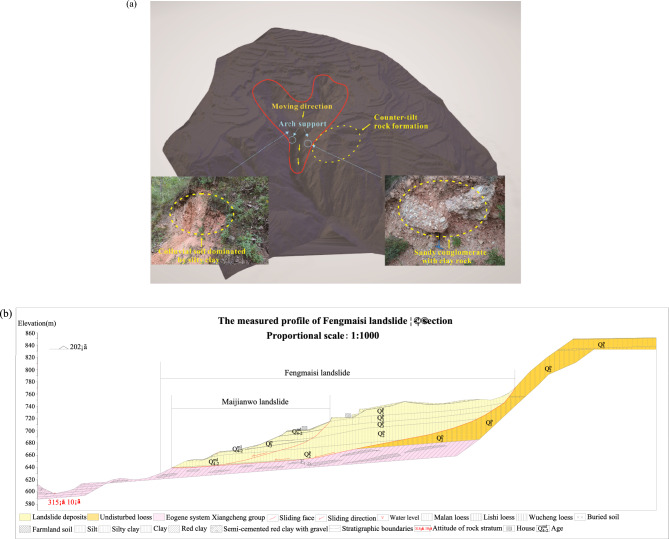
Topographical map of the Maijianwo landslide. (**a**) Overall view of the landslide. (**b**) Longitudinal profile of the landslide study area.

The Maijianwo landslide is located in Fengmaisi Village, Wumu Township, Lingbao City, and is a medium-sized potential loess landslide developed on the Fengmaisi landslide. The landslide is a new landslide which first slid with Fengmaisi landslide in 1964 and resurrected in 2004, which has been consolidated and compacted for a long time and is now in a basically stable state^10,21^. The three-dimensional model of Maijianwo landslide is obtained by UAV aerial photogrammetry and later three-dimensional model reconstruction (see Fig. 2a). Longitudinal profile of the landslide study area is modified on the basis of the geological environment survey report (see Fig. 2b). Its landslide perimeter is obvious, with a wide top and narrow bottom planform and a spoon shape (Fig. 2a). The landslide is about 240 m long, 120 m wide at the top and 40 m wide at the bottom, with a thickness of 15 m. According to the analysis of field survey and exploration data, the overall tendency of the sliding surface is high in the south west and low in the north east, and it is a slowly inclined arc with a tendency to the north east (Fig. 2). The sliding body is located in the middle of the landslide, and there is a nearly north–south gully on both sides of the east and west sides of the sliding body, which merges with the two gullies (dry gullies) in the front edge of the main sliding body and enters the muddy ditch northward (Fig. 2a). Therefore, the sliding body laterally loses connection with the original slope, which makes the sliding body itself has a relatively greater tendency to slide. The obvious sliding occurred in the middle and rear of the sliding body, but it has not yet been destabilized as a whole, constrained by the spoon topography of the leading edge.

According to the analysis of field investigation and exploration data, it is highly likely that there is a horizontal sustaining arch across the landslide at the narrowing of the sliding zone of the Maijianwo landslide. The slip path here becomes narrow, and there is a large amount of high stone content of gravel soil accumulation (Fig. 2a). The west side of the sliding body is located at the corner of the gully, and there are counter-tilt rock formations (The bedrock tendency is opposite to the slope direction) on the east side. The above unique topographical and geomorphological conditions are very conducive to the formation of sustaining arch. The middle and front sliding surface of this landslide is located near the water table (Fig. 2b), and the soil near and below the water table is often in a loose or saturated state, which not only increases the weight of this section of the landslide, but also reduces the shear strength of the soil near the sliding surface. In the rainy season and heavy rainfall conditions, precipitation penetrates into the ground through joint fissures and tensile cracks at the trailing edge of the landslide. In addition to flush and leaching, it also increases the weight of the entire sliding body and reduces the strength of the anti-sliding section. Under the action of gravity-based natural forces, the possibility of landslide resurrection increase.

### Geological structure characteristics of typical sustaining arch locked-segment-type slopes

Through the above analysis, it is found that the disaster-forming environment and geological structure characteristics of the two typical sustaining arch locked-segment-type slopes are similar. Under the action of unloading, subsidence, corrosion and gravity, the cliff area collapses frequently for a long time, and the deposits accumulate in the gentle slope and groove zone. The continuous accumulation and thickening of slope deposits form the material basis of landslide. The east and west sides of the slope are gullies or faults, one side of which is narrow at the beam opening, and the other side is at the bend. The front edge is flat and open, with some space to move around (Fig. 2). After the landslide experienced reactivity, obvious tensile cracks appeared at the trailing edge of the landslide. Due to the decrease of potential energy and the increase of leading edge resistance, the leading edge is temporarily stabilized and gradually compacted and consolidated. Its deformation evolution is controlled by the topographic conditions in addition to the deformation and strength characteristics of the geotechnical body. This special topographic form causes the stress to concentrate at the spoon of the landslide and produces the natural soil arching phenomenon, which has a locking control effect on the evolution and development of the landslide^3,8,10,13^. The self-weight of the slope and the rainfall are the main triggering factors for the overall instability of the landslide^36,40,41^. At present, due to the slip resistance of the narrow section and the locking effect of the sustaining arch, the leading edge of the Maijianwo landslide is still in a stable state and no overall instability has occurred. However, according to the analysis of geological environmental conditions, there is a great possibility that the slope will be active again under the influence of artificial activities and rainfall.

## Physical model

### Experimental material

**Figure 3:**
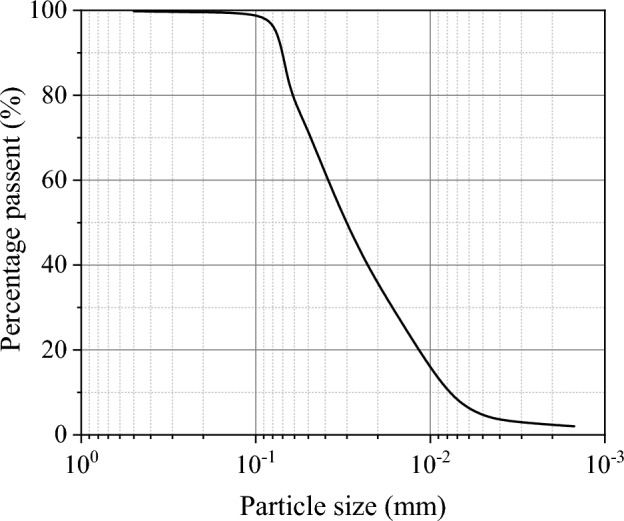
Particle size distribution curve of the silt.

The western region of Henan province is located in the transition zone from the second step to the third step of China’s terrain, which is a landslide-prone area in Henan Province. The basic physical properties of the soil obtained from the western region of Henan province are shown in Table 1. The liquid limit and plastic limit of the sample are 21.4% and 12.6%, respectively, and the plasticity index of the sample is 8.8. As shown in Fig. 3, the clay content of the sample is less than 10%, and the soil sample is sandy silt. The soil specimens used in this experiment had a water content of 15% and a dry density of 1.77 g/cm^3^. The shear strength parameters measured by automatic direct shear apparatus under this condition are shown in Table 1.

**Table 1:** Physical and mechanical properties of the silt.

Specific gravity, *G*_s_	Liquid limit, *w*_L_ (%)	Plastic limit, *w*_P_ (%)	Plastic index, *I*_p_	Maximum dry density, *ρ*_d_ (g*/*cm^3^)	Optimum water content, *w*_opt_ (%)	Cohesion *c *(kPa)	Internal friction angle *φ* (°)
2.70	21.4	12.6	8.8	1.77	15.0	12.8	18.8

### Model test device

**Figure 4:**
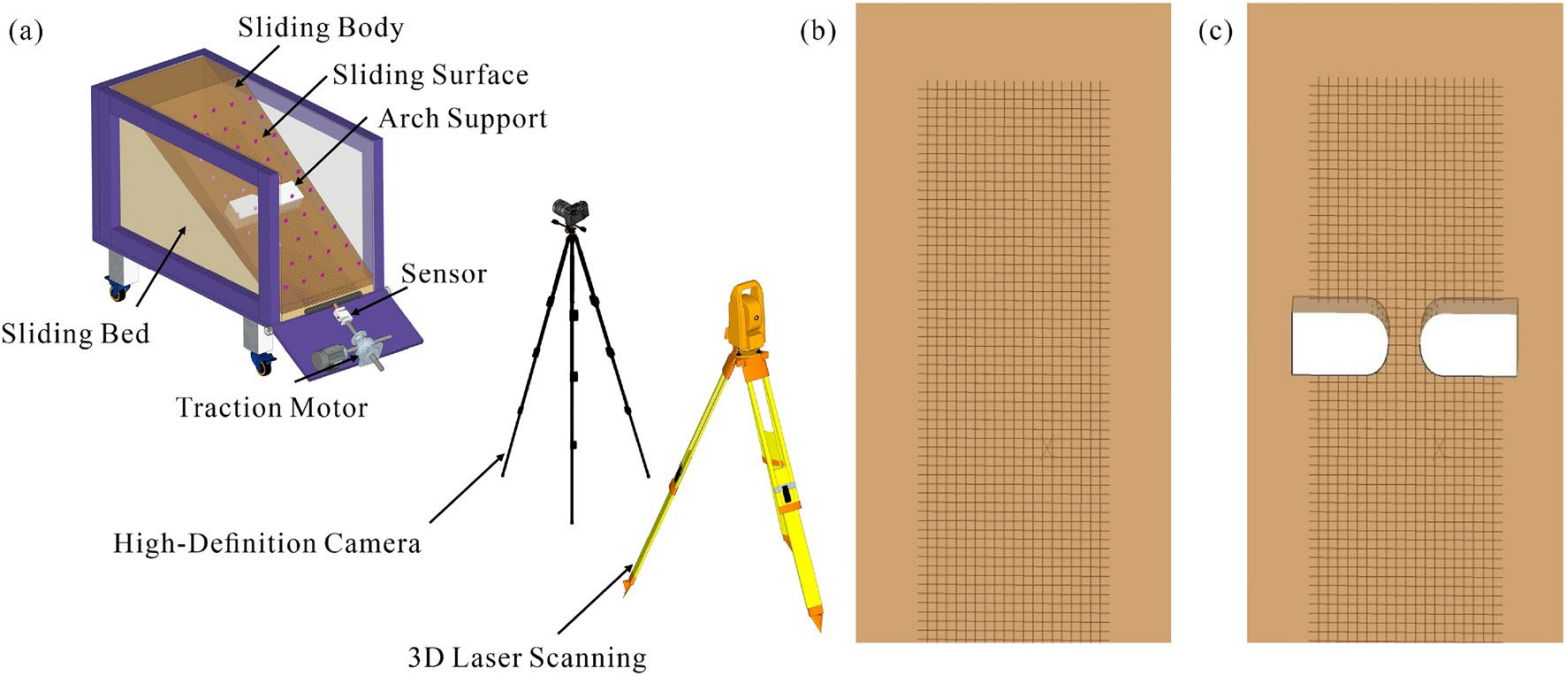
Model test device for accumulation slope. (**a**) Model test system. (**b**) Front view of the slope model without arch support. (**c**) Front view of the slope model with arch support.

The schematic diagram of the self-designed model test device is shown in Fig. 4a, which mainly composed of a Canon high-definition camera, DH3821 stress and strain data acquisition system, and a Leica Nova MS50 three-dimensional laser scanner. The model box's outer dimensions are 120 × 50 × 80 cm, with a steel frame and an organic glass panel on the side wall. The anti-sliding force of the front edge of the slope is measured by the tensile sensor ahead. The tensile sensor used in this experiment is a stress–strain acquisition instrument with a range of − 1000–1000 N, and an accuracy of 0.01 N. A low-speed traction motor is used to pull the tension bars placed on a smooth sliding bed, and the movement of the tension bars drives the soil movement to simulate the traction start-up. By comparing with geomembrane and geogrid, it is found that the chrome-plated iron wire mesh has a uniform force and good traction effect on the soil, and its own force can be more accurately transmitted to the tension sensor. Therefore, the chrome-plated iron wire mesh is selected as a tension bar for traction start-up. The size of the barbed wire mesh is 1.42 × 1.42 cm, the width is 20 cm, and the length is 80 cm. The middle part of the wire mesh is cut off to embed the arch support. During the experiment, the arch support passes through the set sliding surface and penetrates the deep position of the slope. In the experiment, the arch support model (length: 10 cm, width: 6 cm, thickness: 6 cm) is made of formaldehyde-free PVC material, as shown in Fig. 4b.

The slope deformation in the process of slope instability and failure was monitored using a high-definition camera and a three-dimensional laser scanner. The high-definition camera was used to capture the overall deformation of the slope and the progression trend of cracks and soil deformation in different sections of the slope throughout the formation and destruction of several sustaining arches. The movement trend of the soil is recorded using the three-dimensional laser scanner, and the point cloud data is analyzed by software to obtain the deformation field of soil in different regions.

### Model test scheme

Based on the self-designed model test device, a three-dimensional laser scanner and high-definition camera are used to conduct slope tests with various arch spacing (*s*) and without arch support. Considering the size of the model box, three experimental groups with different arch spacing of 5, 7 and 9 cm and the foundation test group without arch support were set up, as shown in Fig. 4.

To facilitate traction start-up, the sliding bed of this physical model test should be hard and smooth. Therefore, the sliding bed in the model box was compacted by rolling compaction in the range of 0–35°. The tensile bars were placed in the same position on the flat surface before each test, at a 35° angle between the tensile bars and the horizontal ground, with the low-speed motor running at 1.5 mm/min. During the test, a high-definition camera recorded the entire process of slope evolution and the formation and destruction of the sustaining arch. The tensile sensor recorded the anti-sliding force curve, and the 3D laser scanner collected the displacement cloud map of the slope surface every two minutes.

## Test results

Three groups of tests with arch spacing (*s*) of 5, 7, and 9 cm were introduced to evaluate the influence of various arch spacing conditions on sustaining arches and slope deformation evolution. To evaluate the influence of the locking effect of the sustaining arch on the stability of the slope, the test group without arch support was chosen as the reference group. The test results were mainly analyzed from the anti-sliding force curve and the evolution of slope deformation.

### Anti-sliding force analysis

**Figure 5:**
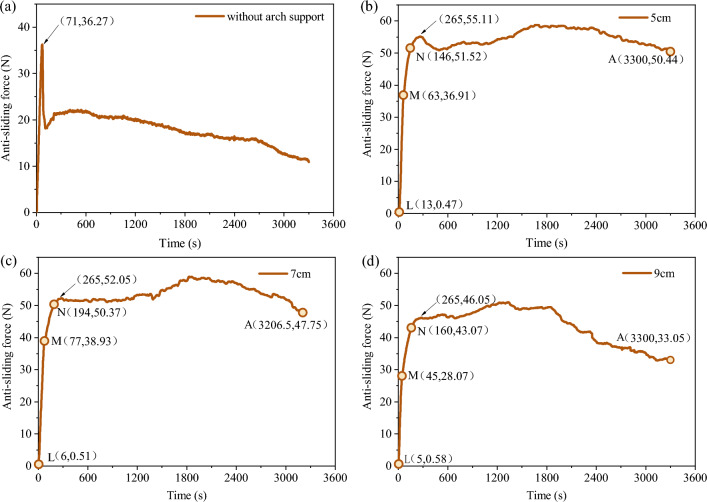
Relationship between anti-sliding force and time. (**a**) without arch support. (**b**) *s* = 5 cm. (**c**) *s* = 7 cm. (**d**) *s* = 9 cm.

As shown in Fig. 5a, the anti-sliding force of the reference group without arch support reaches the peak strength point at 71 s, and the energy accumulated in the earlier stage is fully released, which shows that the anti-sliding force curve decreases rapidly after reaching the peak strength point. It can be seen from Fig. 5b–d that the anti-sliding force of the test group with various arch spacing is significantly higher than that of the test group without arch support. Due to the continuous formation of the natural sustaining arch, the anti-sliding force did not decrease rapidly after reaching the first peak stress point, but continued to adjust the stress to form the next soil arch with stronger locking effect. Combined with the slope deformation evolution process obtained by the high-speed camera and the three-dimensional laser scanning, the anti-sliding force curve of each arch spacing test group can be roughly divided into three stages: LM is the elastic stage, MN is the slope start-up stage, and NA is the continuous action and failure stage of the sustaining arch. The fluctuation of growth and decline of the anti-sliding force curve in the NA stage is related to the formation and failure process of the sustaining arch. And it can be observed that there are three sustaining arch evolutions in the NA stage.

**Figure 6:**
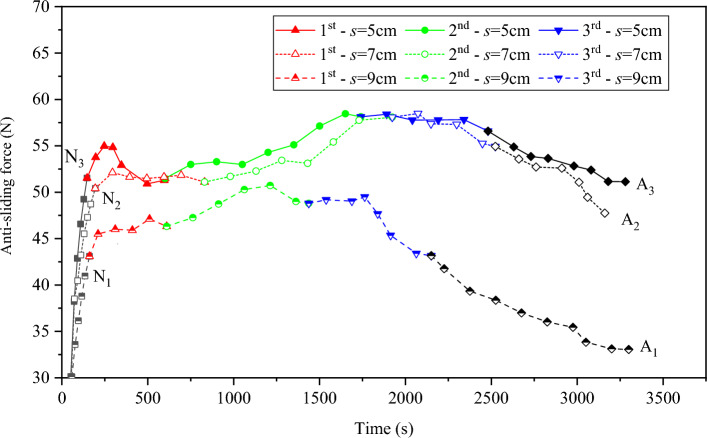
Variation law of anti-sliding force of test groups with various arch spacing conditions.

It can be observed from Fig. 6 that in the action and failure stage of the first sustaining arch (1st arch), the variation law of the anti-sliding force of the test groups with various arch spacing conditions is not the same. The test groups with an arch spacing of 5, 7, and 9 cm show a downward trend, relative stability, and an upward trend, respectively. In the action stage of the second sustaining arch (2nd arch), the anti-sliding force curves of the three groups of tests significantly increased and lasted for a long time, and the anti-sliding force value reached its maximum. At this stage, the locking effect of the sustaining arch was the strongest. The entire locking structure was destroyed after the third sustaining arch (3rd arch) was damaged. The strength of the arch foot of the sustaining arch reached its ultimate shear strength, and the new sustaining arch has cannot be formed in the future. Therefore, the entire locking structure is destroyed. Therefore, in the failure stage of the third sustaining arch (3rd arch), the accumulated energy in the earlier stage was fully released and the sliding force decreased. Through the comparative analysis of the three test groups, it was found that the test groups with 5 and 7 cm arch spacing had a longer continuous action time of the sustaining arch than the test group with 9 cm arch spacing, and their anti-sliding force was also greater than that of the test group with 9 cm arch spacing.

### Comparative analysis of slope deformation evolution

**Figure 7:**
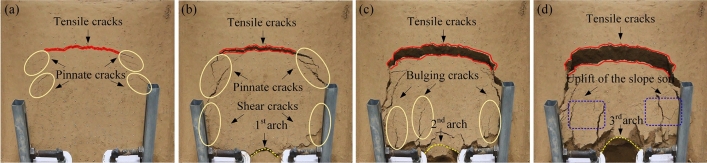
Slope cracks of 9 cm arch spacing test group. (**a**) 160 s. (**b**) 576 s. (**c**) 1348 s. (**d**) 1850s.

**Figure 8:**
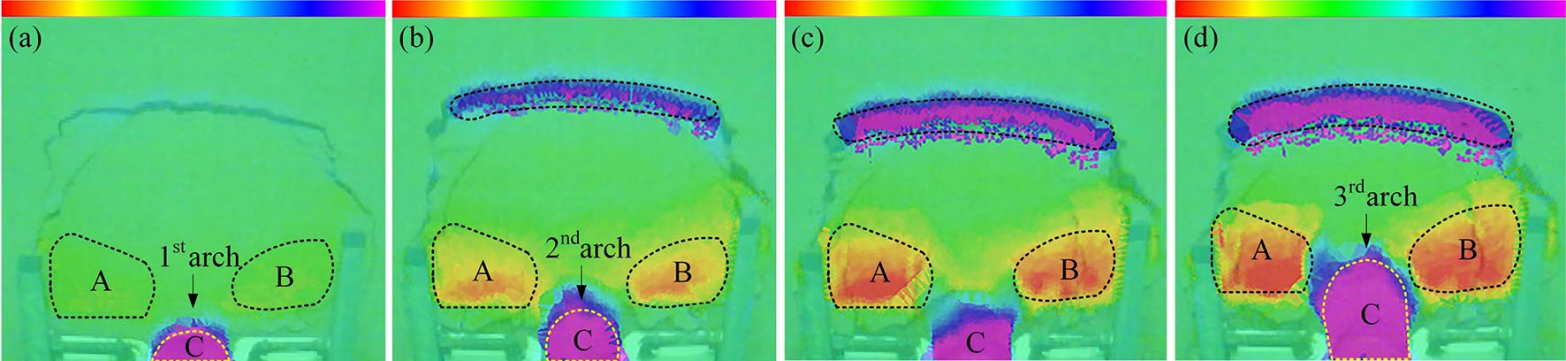
Scanning cloud images of 9 cm arch spacing test group. (**a**) 576 s. (**b**) 1440 s. (**c**) 1800s. (**d**) 1920s.

The continuous formation and progressive failure of the sustaining arch was observed in the deformation evolution process of the test groups with different arch spacing. The overall slope evolution process of the experimental group with 9 cm arch spacing obtained by the high-definition camera is shown in Fig. 7. Three-dimensional laser scanning technology is used to obtain the cloud image, and the cloud image near the node is selected to analyze the slope deformation of the slope evolution process, as shown in Fig. 8. In the cloud image, the purple part represents the soil collapse, and the red part represents the soil uplift. The evolutionary trend and range of the sustaining arch formed between arch supports can be observed emphatically.

During 0–160 s, the slope is in the elastic stage and start-up stage, the surface cracks are less. A local compression phenomenon is formed on the back of the arch supports because of the small relative displacement between the arch supports and soil. At the end of this stage, arch tensile cracks and symmetrical feather cracks occur on both sides at the trailing edge of the slope, as shown in Fig. 7a. During 160–3300 s, the slope is in the stage of continuous failure of the sustaining arch. According to the formation and failure process of the sustaining arch and the change of the anti-sliding force curve (Fig. 6), the slope deformation evolution is divided into four stages.

The first stage corresponds to the 1st sustaining arch (friction arch) action stage (160–576 s). As the slope thrust increases gradually, the soil arch is forced to bear a part of the slope thrust to assist the arch supports against sliding^31^. Then, the friction soil arch is formed, and the initial balance is lost. The compression region on the back of the arch supports is extended, and the friction arch foot is on the arch support side. The pinnate cracks on both sides strengthen at this point, and the circular tensile cracks on the trailing edge penetrate and connect with the shear cracks emerging on the flanks, forming an overall slip boundary, as shown in Fig. 7b. Furthermore, at the end of this stage, the arched crack penetrates and the soil in area C between the arch supports is clearly damaged, as shown in Fig. 8a.

The second stage corresponds to the 2nd sustaining arch (mixed soil arch) action stage (612–1348 s). At this stage, the cracks at the trailing edge of the slope increase and deepen, the soil at the trailing edge sinks, and several bulging cracks appear at the leading edge of the slope. With increasing slope thrust, an end-bearing arch is formed with the back of the arch as the foot support, which is located behind the friction arch to compensate for the lack of bearing capacity of the friction arch. Therefore, the larger arch cracks appear in the upper part of the previous arch ring.

The third stage corresponds to the 3rd sustaining arch (mixed soil arch) action stage (1463–1850 s). The displacement of soil between the arch supports continues to increase in the end-bearing arch area as compression density increases. Owing to the low bearing capacity of the friction soil arch, when the dislocation displacement of the side wall of the arch supports and the soil between the supports is large, the friction soil arch foot is damaged, and the friction soil arching effect gradually weakens or even fails. At this stage, the trailing edge of the slope sinks rapidly, and the slope wall is obviously exposed, as shown in Fig. 7d. As shown in Fig. 8c, it can be observed that the upper A and B regions of the arch supports are the most seriously uplifted. At the end of this stage, the soil below the end-bearing arch falls, and the soil near the arch supports also falls locally, as shown in Fig. 7d.

The fourth stage corresponds to the failure stage of the locking effect of the sustaining arch (2150–3300 s). When the arch foot reaches its ultimate shear strength, the soil arch undergoes shear failure. The soil around the arch supports is extruded and flows around in a large range, and the support system of the sustaining arch fails. The left and right uplift parts are gradually separated from the surrounding soil and become independent blocks attached to the slope, as shown in Fig. 13a.

### Comprehensive analysis of the test results

**Figure 9:**
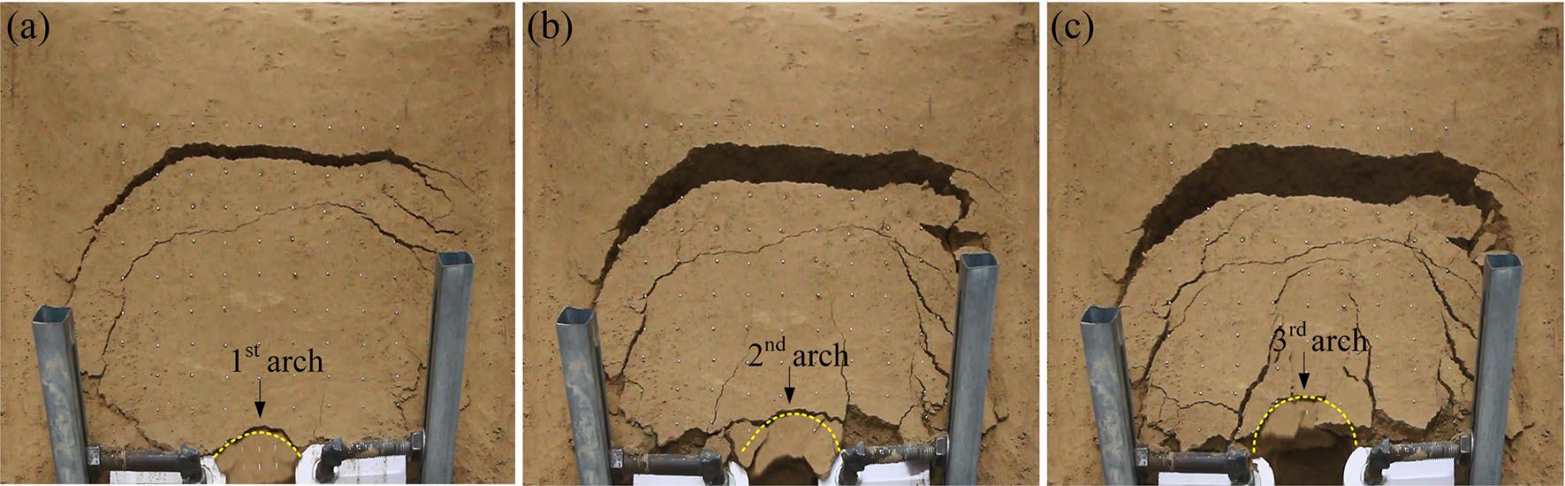
Slope cracks of 7 cm arch spacing test group. (**a**) 830 s. (**b**) 1876s. (**c**) 2410 s.

**Figure 10:**
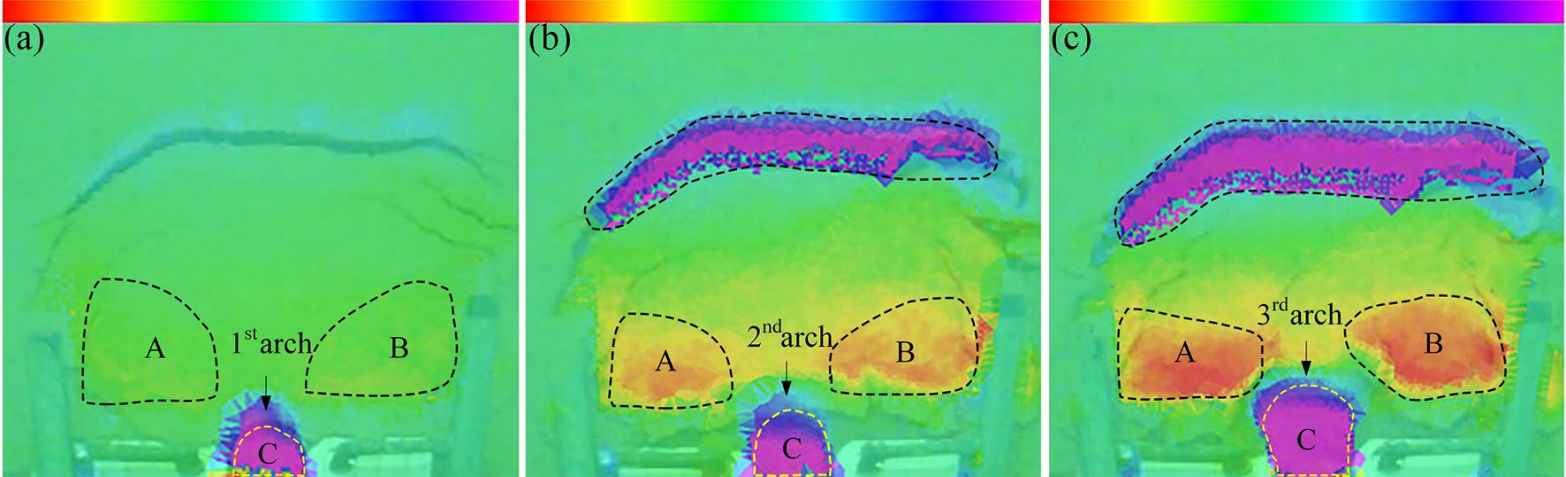
Scanning cloud images of 7 cm arch spacing test group. (**a**) 840 s. (**b**) 1860s. (**c**) 2460 s.

**Figure 11:**
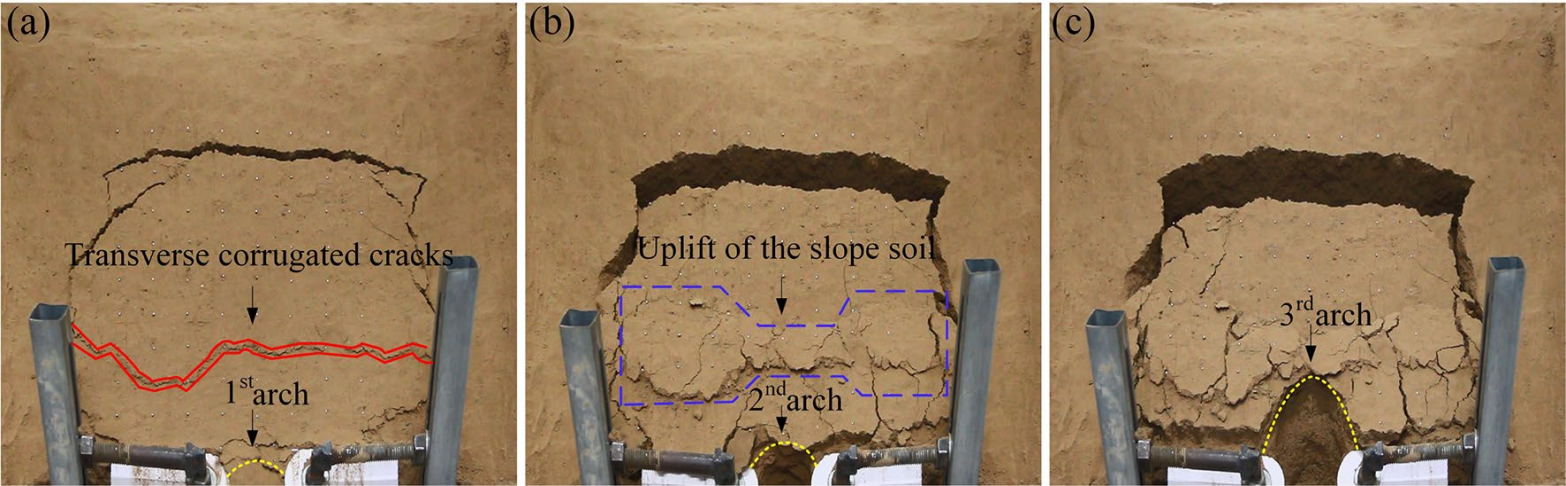
Slope cracks of 5 cm arch spacing test group. (**a**) 570 s. (**b**) 1710s. (**c**) 2435 s.

**Figure 12:**
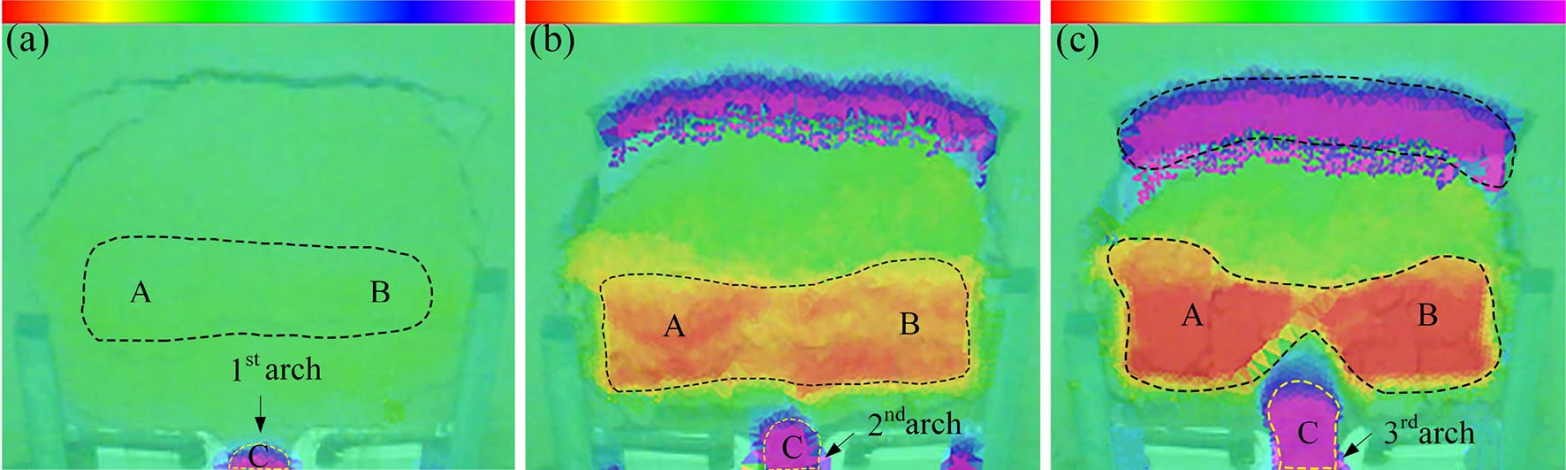
Scanning cloud images of 5 cm arch spacing test group. (**a**) 600 s. (**b**) 1740s. (**c**) 2460 s.

**Figure 13:**
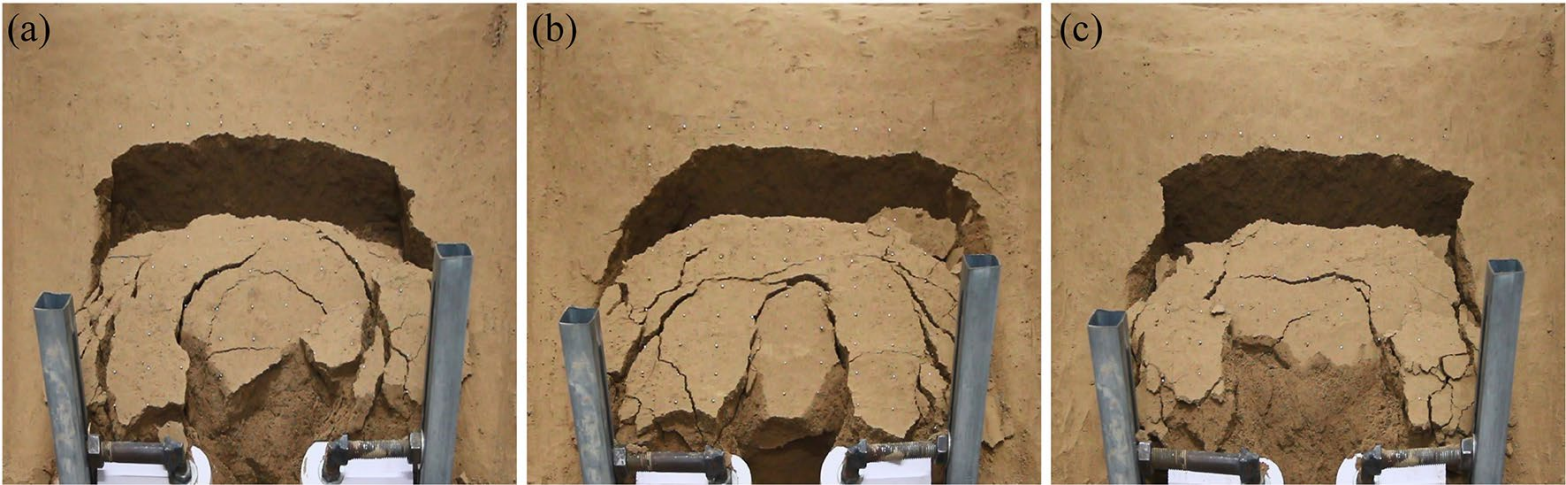
Slope deformation at the end of the three groups of tests. (**a**) *s* = 9 cm. (**b**) *s* = 7 cm. (**c**) *s* = 5 cm.

Through comprehensive comparative analysis of the experimental phenomena of three groups of various arch spacings (Figs. 7, 8, 9, 10, 11, 12), it can be found that the information reflected by the slope is similar. The differences are listed as follows. (1) As the arch spacing of the 5 cm arch spacing test group was too small, the soil was severely squeezed, and the transverse corrugated cracks running through the slope boundary appeared in the middle and lower parts of the early slope (Fig. 11a). In the early stages of 7 and 9 cm test groups, no large crack was observed on the slope surface within the slope perimeter. (2) As arch spacing decreased, the uplift area gradually expanded to the middle channel (Figs. 8c, 10b and 12b). The uplift area with an arch spacing of 5 cm was the most extensive, which spread across the entire slope (see Fig. 13c). In addition to the failure of the sustaining arch between the channels and the falling of the soil near the arch support, the other parts of the 7 and 9 cm arch spacing test groups did not undergo soil sliding phenomenon, as shown in Fig. 13a and b.

In all three sets of experiments, the formation of the sustaining arch is continuous and the failure is progressive. With the increase of the arch height, the formation and destruction of the sustaining arch have gone through three processes. The sustaining arch formed in the second time has the best locking effect, and the anti-sliding force reaches its stress peak point. However, the slope is not in a critically unstable state, instead, the stress is continuously adjusted to form a larger range of soil arch to resist the slope thrust. Until the foot of the sustaining arch reached its ultimate shear strength, the arch ring collapsed and a new soil arch could no longer be formed and the slope destabilized, at which point the arch height reached its maximum.

## Locking effect and critical arch height of the sustaining arch

The formation of the soil arching effect requires the relative displacement of the soil under the action of force, and the support of the stable arch foot and the shear strength of the soil are not less than the maximum shear force^42,43^. From the aforementioned analysis, three sustaining arches were formed during the experiment. When the arch foot of the last arch is damaged, the vector height of the soil arch reaches the critical height, and the locking effect of the sustaining arch fails, and the slope slides as a whole. Therefore, the concept of critical vector height can be proposed to predict the overall sliding time of the slope for sustaining arch locked-segment-type slopes that can form multiple sustaining arch structures. That is, when the critical arch vector height is reached, the ultimate shear strength of the arch foot on the slope is destroyed, and the surrounding soil flows around the arch. Thus, the sustaining arch retaining system fails. It can be inferred from the previous analysis that the soil arch of the last locking effect is a mixed soil arch. According to the mechanical calculation model of the sustaining arch, the corresponding calculation equation of the critical arch height is derived by considering the control conditions of the static equilibrium between the arch and the strength of the arch foot.

When there are hard arch supports in the slope, the soil behind the arch supports is constrained by the arch supports to form a compaction area. Because of the lack of support, the soil between the arch supports is displaced and constantly tightened by the action of the slope thrust, causing the slope thrust borne by it to continue to expand to both sides until it extends to the rear compaction area of the arch supports, forming an arch-shaped stress structure, that is, the sustaining arch. The sustaining arch mentioned here is different from the arch structures such as bridges. The arched structure first has the arch shape and then has the bearing capacity. The formation of the sustaining arch is the result of uneven displacement within the soil under the action of force, and the strength of the soil is adjusted to resist the external force through its own characteristics^43^. Therefore, the force developed leads to the appearance of the arch^23^. Soil arching with a reasonable arch axis occurs only under pressure and the tensile properties of soil are almost nonexistent. Therefore, it is reasonable to assume that the maximum bearing capacity of the soil arch can only be achieved under reasonable conditions of the arch axis^28,35,44^.

### Locking effect and height-span ratio of the sustaining arch

**Figure 14:**
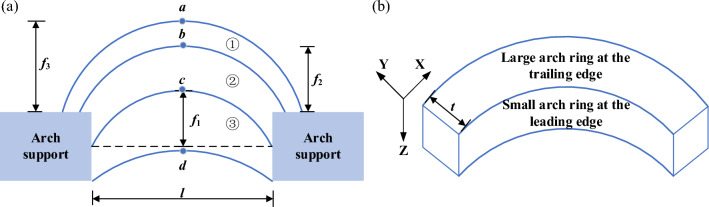
Schematic diagram of the sustaining arch form. (**a**) Distribution of the three arched cracks in the test. (**b**) Sustaining arch of unit height.

There are (1) ~ (3) evolutions of three sustaining arches in the three groups, and the occurrence order of arched cracks in each group gradually expands from the arch supports to the back of the arch supports. As shown in Fig. 14a, the arch lines *a*, *b*, and *c* represent the three arched cracks in the experiment. The unit length of the sustaining arch along the vertical slope direction is shown in Fig. 14b. The sustaining arch has a large rear arch ring and a small front arch ring. Large and small arch rings cannot be simultaneously observed during the action of the sustaining arch, but arched cracks (*a*, *b*, *c*) can be clearly observed when they are damaged and disintegrated. This is the position of the large arch at the rear edge of the damaged sustaining arch and also the position of the small arch at the front edge of the next sustaining arch. As the falling range of the 1^st^ sustaining arch comprises all areas below crack c, the leading edge small arch ring d cannot be observed in the test. That is, $f_{1}$, $f_{2}$, and $f_{3}$ are the vector heights of the cracks at the trailing edge of the 1st, 2nd, and 3rd sustaining arches, respectively.

**Figure 15:**
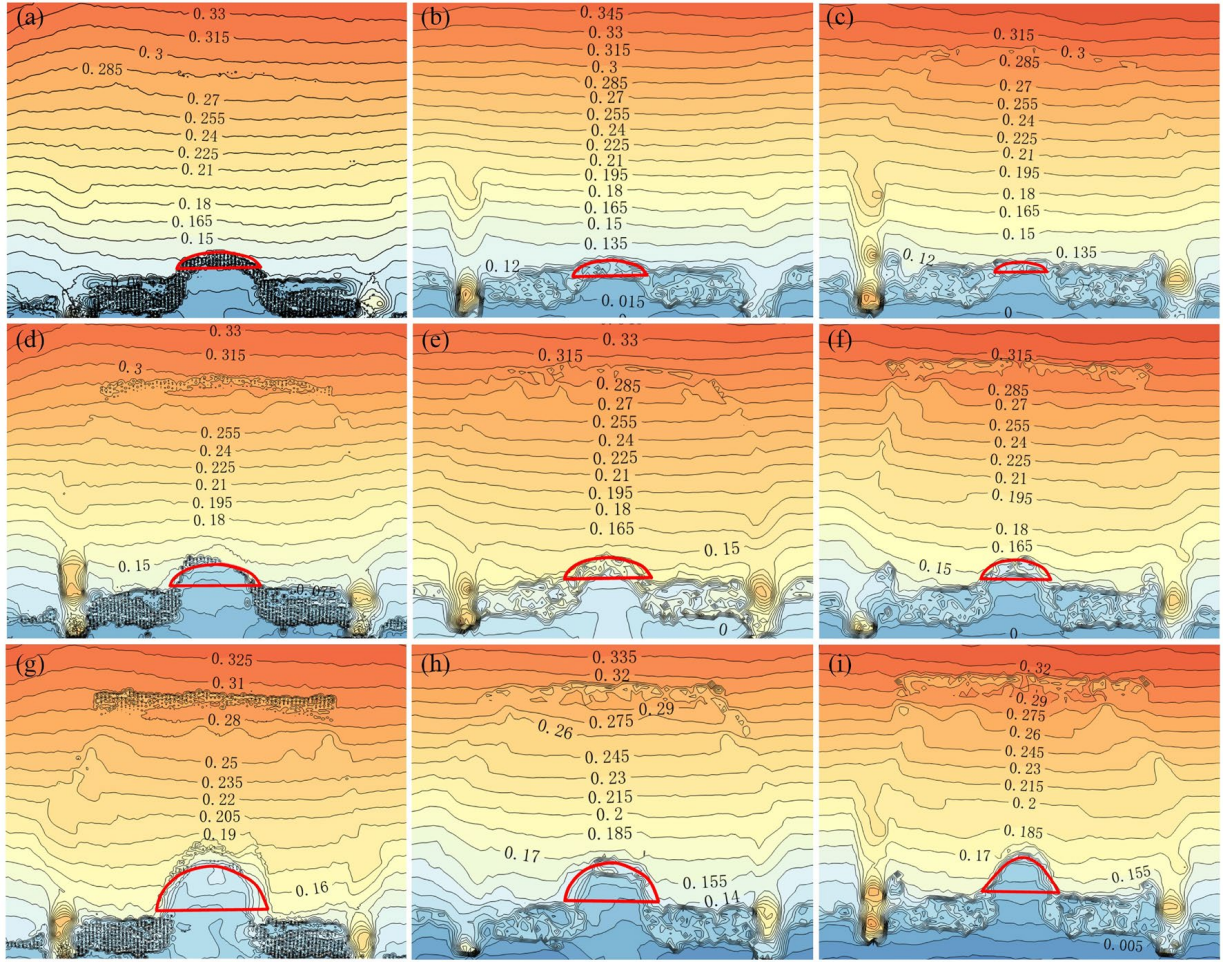
Slope deformation field at failure time of the sustaining arch with various arch spacing conditions. (**a**) *s* = 9 cm-1st arch. (**b**) *s* = 7 cm-1st arch. (**c**) *s* = 5 cm-1st arch. (**d**) *s* = 9 cm-2nd arch. (**e**) *s* = 7 cm-2nd arch. (**f**) *s* = 5 cm-2nd arch. (**g**) *s* = 9 cm-3rd arch. (**h**) *s* = 7 cm-3rd arch. (**i**) *s* = 5 cm-3rd arch.

The point cloud data of the slope surface obtained by the three-dimensional laser scanner during the experiment was processed by Suffer15 to generate the contours of the slope deformation field with digital information (as shown in Fig. 15). The evolution of the three arched cracks can be seen in the figure, and the arch height gradually extends backward as the test progresses. In the software, the center distance *l* and vector height *f* of the arched cracks after each destroyed sustaining arch are measured, and the height-span ratio *f*/*l* is calculated. The test results are shown in Table 2. The vector height of the sustaining arch shown in the table corresponds to the vector height of the large arch ring at the trailing edge and the vector height of the small arch ring at the leading edge of the next sustaining arch.

**Table 2:** Height-span ratio data with various arch spacing conditions.

Parameters	*s* (cm)								
	9			7			5		
Arched crack	1st	2nd	3rd	1st	2nd	3rd	1st	2nd	3rd
*f* (cm)	1.9	2.6	5.0	1.8	2.4	4.3	1.2	2.0	4.0
*l* (cm)	10	11	13	9	10	11	6	8	9
*f*/*l*	0.19	0.24	0.38	0.2	0.24	0.39	0.2	0.25	0.44

It can be observed from Table 2 that when the 1st sustaining arch was damaged, slope instability did not occur. The soil readjusts the internal structure to form the next sustaining arch, and the vector height increases compared with that of the previous sustaining arch. In theory, as long as the arch foot is not damaged, it can always provide a stable and solid foothold for the sustaining arch. That is, as long as the soil can pass through the narrow channel between the arch supports, the locking effect of the sustaining arch can repeatedly occur, and the vector height of the arch increases continuously. However, during the test, the arch foot of the slope was completely destroyed because it reached the ultimate shear strength, and the soil arch height reached the critical height. No new sustaining arch could be formed, and the locking effect of the sustaining arch failed, and the slope slid as a whole.

### Mechanical calculation model of the sustaining arch

In the study of soil arching effect, the soil arch can be simplified as an arch beam for stress analysis, which is called arch beam method. This method is used to analyze the stress of the sustaining arch. To simplify the calculation, the following reasonable assumptions are proposed^45^.

**Figure 16:**
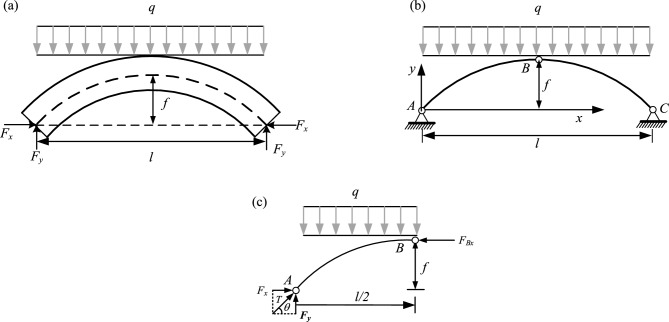
Stress plane diagram of the soil arch. (**a**) Mechanical calculation model of the soil arch. (**b**) Simplified calculation model of the soil arch. (**c**) Stress analysis diagram of the left half arch AB.

1. The slope thrust of the soil behind the arch is evenly distributed along the arch span, without considering its gradual weakening under the locking effect of the sustaining arch.
2. The soil arch can be simplified to a three-hinged arch, as shown in Fig. 16. It is assumed that the soil arch axis is a reasonable arch axis and is parabolic.
3. Mohr–Coulomb strength criterion is applicable to this model.

Based on the aforementioned assumptions, the mechanical calculation model of the soil arch is established in conjunction with the three-hinged arch theory (see Fig. 16a). In Fig. 16a, q denotes the uniform slope thrust on the unit height of the soil arch, *t* is the thickness of the soil arch, *l* is the net distance between the arch supports, and *f* is the height of the soil arch. The arch foot is subjected to horizontal and vertical support forces (*F*_x_ and *F*_y_), respectively, and a rectangular coordinate system is established (see Fig. 16b). Under uniform vertical load, the reasonable arch axis of the three-hinged arch is a parabola. The parabolic equation is assumed as follows:

1 $$y = ax^{2} + bx + c$$

Substituting the points A (0,0), B (*l*/2, *f*), and C (*l*, 0) into the equation, the solution can be obtained as: *a* = − 4f/*l*^2^, *b* = 4f/*l*,* c* = 0. The axial equation of the soil arch can be expressed as:

2 $$y = - \frac{4f}{{l^{2} }}x^{2} + \frac{4f}{l}x$$

According to the theory of the three-hinged arch, it is known that after the formation of the soil arch, any arch section is not subjected to bending moment and shear force, only subjected to axial pressure. Therefore, the damage of the soil arch is controlled by the compressive strength of the arch section. As shown in Fig. 16c, the left half arch AB is considered for stress analysis:

3 $$\sum X = 0 \Rightarrow F_{{\text{x}}} - F_{{{\text{Bx}}}} = 0$$

4 $$\sum Y = 0 \Rightarrow F_{{\text{y}}} - q \cdot \frac{l}{2} = 0$$

5 $$\sum {M_{{\text{A}}} } = 0 \Rightarrow F_{{{\text{Bx}}}} \cdot f - q \cdot \frac{l}{2} \cdot \frac{l}{4} = 0$$

Combined with Eqs. (3)–(5), the solution can be obtained as:

6 $$F_{{{\text{Bx}}}} = \frac{{ql^{2} }}{8f}$$

7 $$F_{{\text{x}}} = \frac{{ql^{2} }}{8f}$$

8 $$F_{{\text{y}}} = \frac{ql}{2}$$

Combined with Eqs. (7) and (8), the resultant force at the arch foot is expressed as follows:

9 $$T = \frac{{ql\sqrt {l^{2} + 16f^{2} } }}{8f}$$

**Figure 17:**
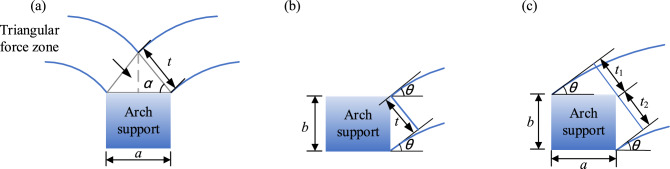
Diagram of the soil arch thickness. (**a**) end-bearing arch. (**b**) frictional arch. (**c**) sustaining arch.

In the equation, *l* is the center distance of the arch (m), *f* is the calculated vector height of the soil arch (m). According to the existing literature, the arch thickness of the soil arch is generally obtained according to the geometric relationship^29^. The thickness diagram of the sustaining arch is shown in Fig. 17. *a* is the effective width of the arch supports facing the soil*,* and *b* is the effective length of the arch support side. From Fig. 17a, it can be inferred that the end-bearing arch behind the arch support has a triangular zone, and therefore, the arch thickness satisfies the equation *t* = *a*/2·cos*α*. From Fig. 17b, it can be inferred that the thickness of the friction arch is *t*_2_ = *b*·cos*θ*. The arch thickness of the sustaining arch model in this test is shown in Fig. 17c, and the arch thickness satisfies the equation *t* = *t*_1_ + *t*_2_. The sustaining arch is different from the end-bearing arch. There is no triangular stress zone on the back of the arch support, and *t*_1_ and *t*_2_ of the arch support can be taken as *a·*sin*θ* and *b*·cos*θ*, respectively, according to the geometric relationship. Thus, the arch thickness can be taken as *t* = *a*·sin*θ* + *b*·cos*θ*.

### Critical arch height

#### Static equilibrium

According to the previous research on the static equilibrium condition of the soil arching effect, to ensure the normal function of soil arch between two adjacent arch supports, it is necessary to meet the static equilibrium condition between arch supports. That is, the sum of the frictional resistance of the sides of the two arch supports should not be less than the pressure acting on the soil arch between the arch supports, it is can be expressed as:

10 $$2(F_{{\text{x}}} \tan \varphi_{s} + tc_{s} ) = ql$$

**Figure 18:**
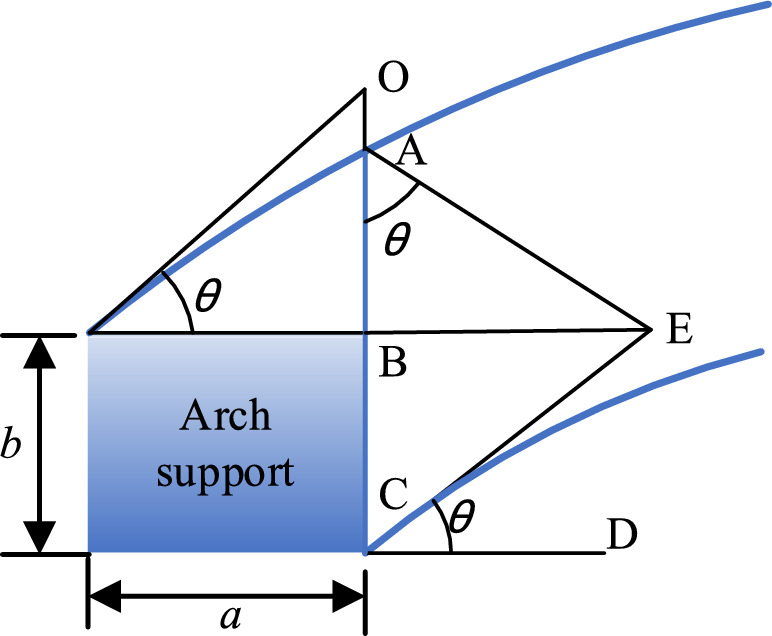
Schematic diagram of the sustaining arch model at the arch support.

However, for the sustaining arch of a mixed arch, the above equation is inadequate to calculate the side friction resistance, and it does not consider the influence of the friction coefficient of the arch support-soil contact surface. Therefore, Eq. (11) is revised to propose the condition that satisfies the static balance of the sustaining arch. As shown in Fig. 18, the sum of frictional resistance on the side of ABC in stable condition should not be less than the pressure acting on the sustaining arch between the arch supports, which can be expressed as:

11 $$2(N_{{{\text{AB}}}} \tan \varphi_{s} + N_{{{\text{BC}}}} \tan \varphi_{f} + L_{{{\text{AB}}}} c_{s} + bc_{f} ) = ql$$

In the equation, *φ*_s_ is the internal friction angle of the soil, and *c*_s_ is the cohesion of the soil. *φ*_f_ is the internal friction angle between the soil on the arch support side and the contact surface of the arch support. When the soil on the side of the arch support consists of a rock or large particles, *φ*_f_ can be taken as *φ*_s_/2. If it is a small particle, *φ*_f_ is taken as *φ*_s_/2 or *φ*_s_2/3. *c*_f_ is the cohesive force between the soil on the arch support side and the contact surface, generally taken as *c*_f_ = *c*_s_·tan*φ*_f_/*φ*_s_. *N*_AB_ and *N*_BC_ are the lateral pressures on the AB and BC surfaces respectively, which can be calculated from the reaction force in the horizontal direction of the arch supports. According to the force characteristics of the three-hinged arch, the lateral pressure can be evenly distributed to the two contact surfaces according to the length of the contact surface.

For the calculation of the length AB, it is usually taken as *a*·sin*θ*_s_ according to previous studies. Although it is easy to calculate, it is equivalent to taking the length of the AO into account. When *θ*_s_ is larger or the arch support length (*a*) is larger, the error increases.

The calculation of the length AB is improved here. According to the geometric relationship, *L*_AB_ = *L*_BF_ /tan*φ*_s_ and *L*_BF_ = *b* /tan*θ*_s_, then *L*_AB_ = *b* /(tan^2^*θ*_s_). The horizontal reaction force of the arch support is 8f/*ql*^2^ obtained from Eq. (7), then the lateral pressure of AB and BC contact surface can be expressed as:

12 $$N_{{{\text{AB}}}} = \frac{{ql^{2} }}{{8f(1 + \tan^{2} \theta_{{\text{s}}} )}}$$

13 $$N_{{{\text{BC}}}} = \frac{{ql^{2} \tan^{2} \theta_{{\text{s}}} }}{{8f(1 + \tan^{2} \theta_{{\text{s}}} )}}$$

The expression for *f* can be obtained from substituting *L*_AB_ = *b* /(tan^2^*θ*_s_), Eqs. (12) and (13) into Eq. (11):

14 $$f_{{1}} = \frac{{ql^{2} \tan^{2} \theta_{{\text{s}}} (\tan \varphi_{{\text{s}}} + \tan^{2} \theta_{{\text{s}}} \tan \varphi_{{\text{f}}} )}}{{4(1 + \tan^{2} \theta_{{\text{s}}} )\left[ {\tan^{2} \theta_{{\text{s}}} (ql - 2bc_{{\text{f}}} ) - 2bc_{{\text{s}}} } \right]}}$$

In the limit equilibrium state, *θ*_s_ = 45° + *φ*_s_/2.

#### Arch foot strength

**Figure 19:**
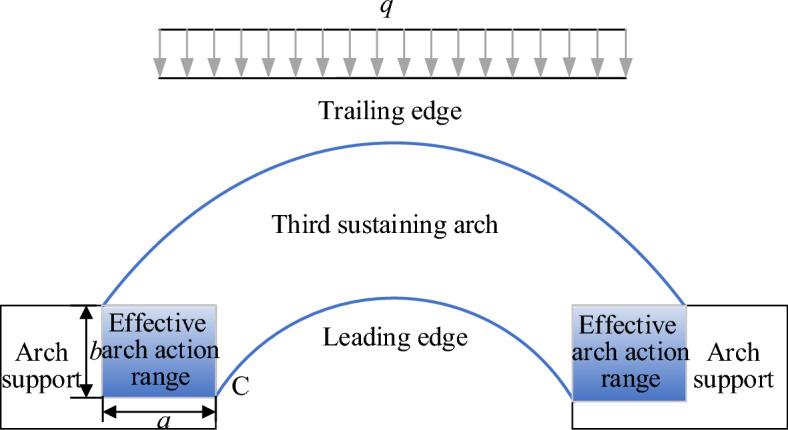
Force diagram on the foot of the sustaining arch.

The soil arch is subjected to the largest forces at the foot of the arch on both sides under the action of the downward thrust^28,29^. Therefore, when conducting a force analysis of the soil arch, it is important to focus on the shear strength at the foot of the arch. For the arch foot section, the leading edge point is more prone to damage than the trailing edge point. Therefore, it is considered that the strength check is carried out according to the Mohr–Coulomb rule at point C (shown in Fig. 19) at the leading edge of the arch foot section, expressed as:

15 $$\sigma_{1} = \sigma_{3} \tan^{2} \theta_{{\text{s}}} + 2c_{{\text{s}}} \tan \theta_{{\text{s}}}$$

The force of the soil between the arch supports on the arch foot C is small, and *σ*_3_ = 0 can be taken. It is considered that the thrust of the slope after the sustaining arch is fully exerted on the sustaining arch. That is, there is no shear force and bending moment in the section of the sustaining arch, only the principal stress acts. Considering that the principal stresses are uniformly distributed along the section, which can be expressed as:

16 $$\sigma_{{1}} = T/t$$

Thereby, substituting *t* = *a*·sin*θ*_s_ + *b*·cos*θ*_s_ and Eq. (9) into Eq. (16), it is obtained as:

17 $$\frac{{ql\sqrt {l^{2} + 16f^{2} } }}{8f \cdot t} = 2c_{{\text{s}}} \tan \theta_{{\text{s}}}$$

Reorganizing the above equation, the critical arch height can be obtained as:

18 $$f_{2} = \frac{{ql^{2} }}{{4\sqrt {16c_{{\text{s}}}^{2} \tan^{2} \theta_{{\text{s}}} (a\sin \theta_{{\text{s}}} + b\cos \theta_{{\text{s}}} )^{2} - q^{2} l^{2} } }}$$

According to the above control conditions, the critical arch height can be obtained under the same stress *q*, and finally, the minimum value is taken as the calculation result, expressed as:

19 $$f = \min \left\{ {f_{1} ,f_{2} } \right\} = \min \left\{ \begin{gathered} \frac{{ql^{2} \tan^{2} \theta_{{\text{s}}} (\tan \varphi_{{\text{s}}} + \tan^{2} \theta_{{\text{s}}} \tan \varphi_{{\text{f}}} )}}{{4(1 + \tan^{2} \theta_{{\text{s}}} )\left[ {\tan^{2} \theta_{{\text{s}}} (ql - 2bc_{{\text{f}}} ) - 2bc_{{\text{s}}} } \right]}}, \hfill \\ \frac{{ql^{2} }}{{4\sqrt {16c_{{\text{s}}}^{2} \tan^{2} \theta_{{\text{s}}} (a\sin \theta_{{\text{s}}} + b\cos \theta_{{\text{s}}} )^{2} - q^{2} l^{2} } }} \hfill \\ \end{gathered} \right\}$$

In the limit equilibrium state, *θ*_s_ = 45° + *φ*_s_/2.

For sustaining arch locked-segment-type slopes, the arch height of the arch crack can be used to determine whether the slope is stable. Although soil sliding and arch structure destruction processes occurs in the early stage of the slope, the slope is still in the limit equilibrium state before reaching the critical arch height, and therefore, the overall slide does not occur.

The actual uniform load acting over the sustaining arch (*q*) is calculated as follows^12^:

20 $$q_{{{\text{ar}}}} = \frac{L}{S}\frac{Q}{P}(1 - e^{ - Pt} )$$

In which:

21 $$K_{0} = 1 - \sin \varphi$$

22 $$K_{{\text{a}}} = \tan^{2} (45^\circ - \varphi /2)$$

23 $$K_{{\text{w}}} = 1.06(\cos^{2} \theta^{\prime } + K_{{\text{a}}} \sin^{2} \theta^{\prime } )$$

24 $$P = \frac{{2K_{{\text{w}}} }}{L}\cos \alpha \tan \varphi_{{\text{f}}}$$

25 $$Q = \gamma \cos \alpha \sin \alpha - \left( {\frac{{\gamma hK_{0} }}{L}\cos \alpha \tan \varphi_{{\text{f}}} + \frac{{2c_{{\text{f}}} }}{L}\cos \alpha + \gamma \cos^{2} \alpha \tan \varphi_{{\text{s}}} + \frac{{c_{s} }}{h}} \right)$$

**Figure 20:**
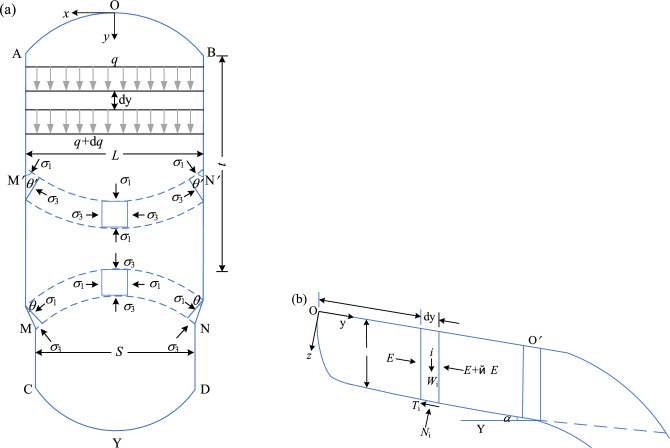
Calculation model of the sustaining arch (modified from references^12^). (**a**) Planar graph. (**b**) Longitudinal profile graph.

In the equation, *t* is the distance from the top of the sustaining arch to the main arm, *L* is the average width of the sliding body, *S* is the span of the sustaining arch, *h* is the depth of the sliding body, and *α* is the inclination of the sliding surface (see Fig. 20). *a* is the width of the compacted part of the soil (i.e., the arch support), *b* is the width of the arch support, and *l* is the distance between the centers of the arch. *γ* is the soil volume weight of the slope and *φ* is the internal friction angle of the soil. *c*_s_ and *φ*_s_ are the shear strength parameters of the sliding surface, respectively. *c*_f_ and *φ*_f_ are the shear strength parameters of the slope flanks, respectively. *θ*′ is the angle between the plane of maximum principal stress action and the slope flank face (rupture plane). *K*_0_ is the mean static earth pressure on the slope flanks and *K*_a_ is the Rankine active earth pressure. *K*_w_ is the lateral pressure coefficient of the soil when the minimum principal stress arch in the slope is formed and the positive stress on the slope flanks is increased because of stress transfer.

## Discussion

### Verification of the physical model test

In order to verify the correctness and applicability of the critical arch height formula, the calculated results are compared with the experimental data of the indoor model for the discussed problem. The basic test conditions of the physical model test in this paper are shown in Table A of the supplementary file. From the experimental results, the cohesion (*c*_s_) and internal friction angle (*φ*_s_) of the silt in western Henan were 10.6 kPa and 18.8°, respectively. The cohesive force (*c*_f_) and internal friction angle between the soil on the arch support side and the contact surface of the arch support (*φ*_f_) were 6.922 kPa and 12.53°, respectively. The depth of the sliding surface (*h*) is considered as the thickness of the arch support, which is 0.06 m. The slope is in ultimate equilibrium before it destabilizes. Therefore, *θ* = 45° + *φ*/2, which is computed to be 54.4°. Considering 7 cm arch spacing test group as an example, the effective width of arch supports facing the soil (*a*) for its last sustaining arch is 0.005 m, the effective length of arch supports side (*b*) is 0.02 m, the arch center distance (*l*) is 0.105 m, the ultimate anti-sliding (*F*) is 57 N, and the uniform load of the slope (*q*) is 9.047 kPa. From Table 2, the vector height at the trailing edge of the second and third arch crack obtained from the model test statistics were 0.024 and 0.043 m, respectively. Substituting the above parameters into Eq. (19), the critical arch height (calculated arch height) of the slope can be obtained as 0.029 m, which is between the vector height at the trailing edge of the second and third arch crack.

**Figure 21:**
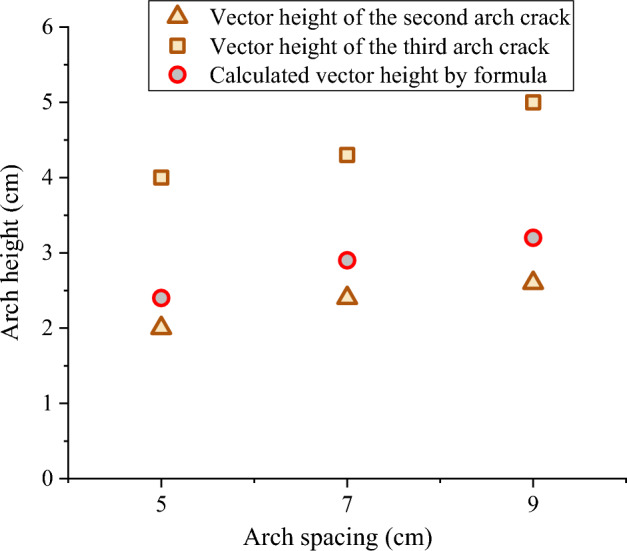
Critical arch height of the soil arch.

Similarly, the same method was used to verify the data of the *s* = 5 cm and *s* = 9 cm test groups. The specific physical parameters obtained from the experimental tests are shown in Table 3. Figure 21 shows that the calculated vector height obtained from the critical arch height equation is between the vector height at the trailing edge of the second and third arch crack. Therefore, combined with the relevant parameters of the slope, the critical arch height of the slope can be obtained by Eq. (19), and the stability of the slope can be determined according to the current arch height of the slope. When the arch height is less than the critical arch height, the slope is stable and can continue to adjust the stress to form the next arch crack. When the arch height is greater than the critical arch height, it means that the arch foot has reached its ultimate shear strength and the slope is about to become unstable.

**Table 3:** Specific physical parameters obtained from the experiments.

*s* (cm)	Parameters									
	*φ*_s_ (°)	*c*_s_ (kPa)	*φ*_f_ (°)	*c*_f_ (kPa)	*h* (m)	*l* (m)	*F* (N)	*q* (kPa)	*a* (m)	*b* (m)
9	18.8	10.6	12.53	6.922	0.06	0.120	51	7.083	0.005	0.027
7						0.105	57	9.047	0.005	0.030
5						0.085	58	11.373	0.005	0.030

### Case study: the Xintan slope

**Figure 22:**
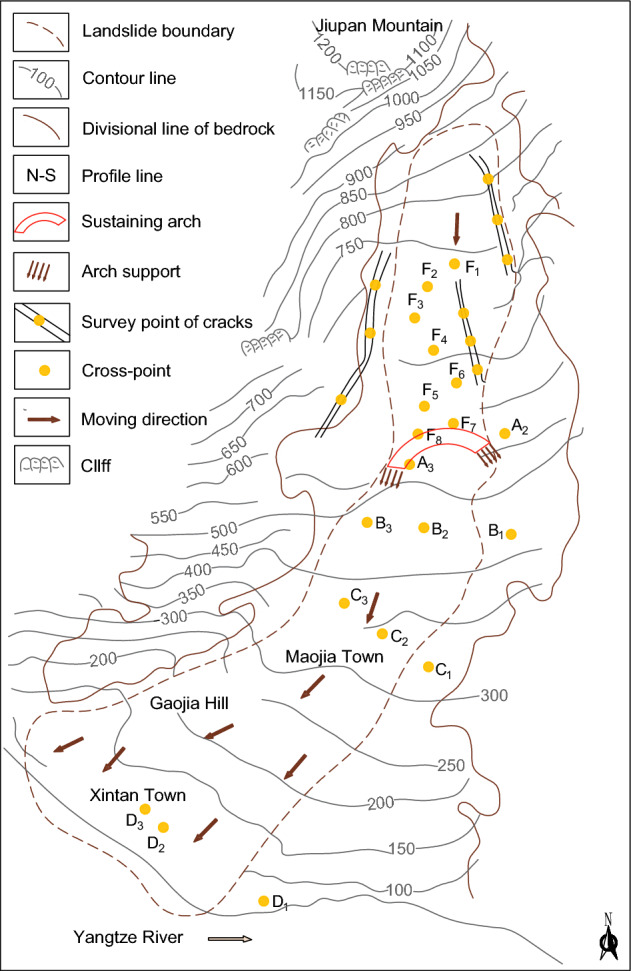
Landform map of the Xintan landslide (modified from references^38,39^).

The destabilization mechanism of Xintan landslide can be summarized as follows (see Fig. 22): under the long-term effect of the self-weight of the slope body and rainfall, the strength of the internal sustaining arch structure of the slope body is gradually degraded. When the damage of the sustaining arch structure evolves to the peak strength point, macroscopic fracture has occurred, resulting in the failure of the current sustaining arch structure locking effect^3^. At this time, slope instability has become inevitable. However, since the foot of the sustaining arch has not yet reached the ultimate shear strength, the slope will not be destabilized and slide immediately. Since then, the slope displacement is significantly sensitive to rainfall. With rainfall infiltration, on the one hand, it increases the slope weight, resulting in increased sliding force^46^. on the other hand, it reduces the shear strength of soil and the contact surface between soil and bedrock. When the arch support and arch foot soil reach the ultimate shear strength, the sustaining arch locking effect fails completely, leading to the occurrence of the Xintan landslide.

According to the geometric characteristics of the Xintan slope^13,38^, the distance from the top of the sustaining arch to the main arm(*t*) is 980 m, the average width of the slide (*L*) is 350 m, the width of the slide at the sustaining arch (*S*) is 300 m, and the depth of the slide (*h*) is 40 m. According to the in-situ geotechnical parameters of the slope site, the width of the compacted part of the soil (*a*) (i.e., the arch support) is 50 m, the width of the side of the arch support (*b*) is 100 m, and the distance between the center of the arch (*l*) is 250 m. The physical parameters of the soil at the critical instability of the slope used are shown in Table 4. Thus, when the slope is in the limit equilibrium state, *θ*′ = 51°, *K*_0_ = 0.58, *K*_a_ = 0.39, and *K*_w_ = 0.67 can be obtained from Eqs. (21)–(23). Substituting the above slope geotechnical parameters into the Eqs. (20), (24)–(25), the mean distributed load above the sustaining arch can be obtained as 47 kPa. By substituting the above critical parameters into Eq. (19), the critical arch height (calculated vector height) of the Xintan slope can be verified to be 70 m. The slope is stable at an arch height of 63 m (vector height at the trailing edge), as analyzed through slope site investigations and indoor physical model simulations^12^. At this time, the arch height of the trailing edge crack is less than the critical calculated arch height, the ultimate shear strength of the arch footing has not yet been reached, and the sustaining arch can continue to induce a locking effect. By adjusting the stress further, a larger range of the soil arch is formed, and the height of the rear edge crack arch exceeds the critical arch height, destroying the soil near the arch foot and the arch support and making the overall landslide unstable. This is consistent with the actual deformation process of the Xintan landslide, which proves the applicability of the critical arch height equation to determine the stability of this type of slope.

**Table 4:** Specific soil physical parameters for critical instability states of slopes.

*γ* (MN/m^3^)	*α* (°)	*φ* (°)	*φ*_s_ (°)	*c*_s_ (kPa)	*φ*_f_ (°)	*c*_f_ (kPa)
0.022	23	25	20	0.028	12	0.016

### Limitations

The natural arch structure discussed in this paper is formed in the deformation evolution process of the supporting arch locking slope. The difference of soil properties in different slope positions, as well as the water content and dry density of soil, have a very important influence on the deformation evolution process. In the future, more influencing factors will be further considered. A critical arch height prediction model for slope stability was validated using the Xintan landslide in China, but still has certain limitations. Firstly, a lot of assumptions were made during the critical arch height modelling process, such as not taking into account the gradual weakening of the slope thrust under the locking action of the sustaining arch. Secondly, the monitoring data on the sustaining arch structure was not incomplete as a result of the early onset of instability in the Xintan landslide, and more such landslide cases need to be investigated to verify the validity of the method. In the future, a series of studies will be conducted to further optimize the proposed method for the prediction and early warning of Maijianwo landslide.

## Conclusion

In this study, the sustaining arch locked-segment-type slope was taken as the research object, and the control group test without arch support and the locked-segment-type slope test with the arch spacing of 9, 7 and 5 cm were set up by using high-definition camera, stress–strain acquisition instrument and three-dimensional laser scanner. The continuous formation and progressive damage of the sustaining arch was discovered. From the perspective of the mechanical mechanism and evolution stage of the slope, considering the influence of the arch support and soil parameters on the soil arching effect, the critical arch height mechanical model for evaluating slope stability is established, which can guide the optimal design of prevention and control engineering of this landslide type. The main conclusions are listed as follows.

1. By comparing the geological environment structure of Xintan slope and Maijianwo landslide, it is found that these two typical sustaining arch locked-segment-type slopes have similar disaster-forming environments. At present, due to the anti-sliding force of the narrow section and the locking effect of the sustaining arch, the leading edge of the Maijianwo landslide has not been significantly deformed and the slope is still in a basically stable state.
2. The slope of the reference test group without arch support reached the peak strength point at 71 s, and then the anti-sliding force curve decreased rapidly. The test group with various arch spacing reached the peak strength point later than the control group, and the anti-sliding force also increased significantly. And due to the continuous formation of the natural sustaining arch, the anti-sliding force did not drop rapidly after reaching the first peak stress point, but continues to adjust the stress to form the next soil arch with stronger locking effect. Therefore, through comparative analysis, it can be concluded that the sustaining arch locking structure has a strong control effect on the slope stability, which improves the slope stability to a certain extent.
3. In test groups with varying arch spacing, the continuous formation and progressive failure process of the supporting arch were observed. And the sustaining arch formed in the second time has the best locking effect, the anti-sliding force reaches its peak stress point at this stage, but the slope is not in a critically unstable state. Instead, the stresses continue to be adjusted to create a wider range of the soil arch. Consequently, the slope destabilizes until the ultimate shear strength of the arch foot is reached, at which point the soil arch reaches a critical arch height.
4. According to the limit equilibrium theory and soil arching effect, the calculation equations of critical arch height under the control conditions are proposed. The critical arch height is related to the arch support parameters, slope rock and soil parameters and sliding body range. The results obtained by the prediction model are consistent with the actual deformation process of the physical model and Xintan landslide, which proves the applicability of the critical arch height equation in maintaining the arch-locked slope and provides a new method for determining the stability of such slopes.

### Supplementary Information

Supplementary Table 1.

## Data Availability

The datasets generated during and/or analysed during the current study are available from the corresponding author on reasonable request.
