# Supplementary material for: Novel evaluation method based on critical arch height as instability criterion for sustaining arch locked-segment-type slopes
Source: Sci Rep. 2024 Apr 5;14:7991. doi: 10.1038/s41598-024-58737-w (PMC10997669; doi:10.1038/s41598-024-58737-w)
Supplement: Supplementary file 1 — Supplementary Table 1. [file 41598_2024_58737_MOESM1_ESM.docx]

**Table A. Summary protocol for the physical model tests**

| Test aim | | Novel evaluation method based on critical arch height as instability criterion for sustaining arch locked-segment-type slopes | | | |
| --- | --- | --- | --- | --- | --- |
| Basic | Triggers | Traction | Container | Model size (cm) | Length:120;  width: 50;  height: 80 |
|  | Landslide  Classification | Silt landslide |  | Preparation | Compaction |
|  | | |  | | |
| Slope model | Angle | 35° | arch support model | Material | PVC material |
|  | Material | sandy silt |  | Arch support size (cm) | length: 10, width: 6,  thickness: 6 |
|  | Properties | *G*_s_: 2.70  *w*_opt_: 15% |  | Arch spacing (cm) | 5, 7, 9 |
|  | | | | | |
| Monitoring  tool | Stress sensor | DH3821 stress and strain data acquisition system | Test  condition | Test cases | 3 |
|  | Point cloud acquisition equipment | Leica Nova MS50 three-dimensional laser scanner |  | Test variable | Arch spacing |
|  | | | | | |
| Important results | | | | | |
| ① | The continuous formation and progressive destruction of the sustaining arch were observed. | | | | |
| ② | The slope does not reach a critical instability at the peak stress point of the highest anti-sliding (2^nd^ arch action stage), but can continue to adjust the stress to form the next soil arch. | | | | |
